# Biodiversity conservation gaps in the Brazilian protected areas

**DOI:** 10.1038/s41598-017-08707-2

**Published:** 2017-08-22

**Authors:** Ubirajara Oliveira, Britaldo Silveira Soares-Filho, Adriano Pereira Paglia, Antonio D. Brescovit, Claudio J. B. de Carvalho, Daniel Paiva Silva, Daniella T. Rezende, Felipe Sá Fortes Leite, João Aguiar Nogueira Batista, João Paulo Peixoto Pena Barbosa, João Renato Stehmann, John S. Ascher, Marcelo Ferreira de Vasconcelos, Paulo De Marco, Peter Löwenberg-Neto, Viviane Gianluppi Ferro, Adalberto J. Santos

**Affiliations:** 1 Centro de Sensoriamento Remoto, Instituto de Geociências, Universidade Federal de Minas Gerais – UFMG, Av. Antonio Carlos 6627, CEP 31270-901 Belo Horizonte, MG Brazil; 2 Departamento de Zoologia, Instituto de Ciências Biológicas, Universidade Federal de Minas Gerais – UFMG, Av. Antonio Carlos 6627, CEP 31270-901 Belo Horizonte, MG Brazil; 3 Departamento de Biologia Geral, Instituto de Ciências Biológicas, Universidade Federal de Minas Gerais – UFMG, Belo Horizonte, MG Brazil; 4 Laboratório Especial de Coleções Zoológicas, Instituto Butantan, São Paulo, SP Brazil; 5 Departamento de Zoologia, Universidade Federal do Paraná, Curitiba, Paraná Brazil; 6 Instituto Federal Goiano – IFGoiano, Departamento de Biologia, Urutaí, Goiás Brazil; 7 Sección Palentología de Vertebrados Museo Argentino de Ciencias Naturales “Bernardino Rivadavia” Avenida Angel Gallardo 470, C1405DJR Buenos, Aires Argentina; 8 Laboratório Sagarana, Instituto de Ciências Biológicas e da Saúde, Universidade Federal de Viçosa – UFV, Campus Florestal, Florestal, MG Brazil; 9 Departamento de Botânica, Instituto de Ciências Biológicas, Universidade Federal de Minas Gerais – UFMG, Belo Horizonte, MG Brazil; 10 Department of Biological Sciences, National University of Singapore, Singapore, Singapore; 11 Instituto Prístino, Rua Santa Maria Goretti, 86, Barreiro, CEP 30642-020 Belo Horizonte, MG Brazil; 12 Departamento de Ecologia, Instituto de Ciências Biológicas, Universidade Federal de Goiás, Goiânia, Goiás Brazil; 13 Universidade Federal da Integração Latino-Americana, Foz do Iguaçu, PR Brazil

## Abstract

Although Brazil is a megadiverse country and thus a conservation priority, no study has yet quantified conservation gaps in the Brazilian protected areas (PAs) using extensive empirical data. Here, we evaluate the degree of biodiversity protection and knowledge within all the Brazilian PAs through a gap analysis of vertebrate, arthropod and angiosperm occurrences and phylogenetic data. Our results show that the knowledge on biodiversity in most Brazilian PAs remain scant as 71% of PAs have less than 0.01 species records per km^2^. Almost 55% of Brazilian species and about 40% of evolutionary lineages are not found in PAs, while most species have less than 30% of their geographic distribution within PAs. Moreover, the current PA network fails to protect the majority of endemic species. Most importantly, these results are similar for all taxonomic groups analysed here. The methods and results of our countrywide assessment are suggested to help design further inventories in order to map and secure the key biodiversity of the Brazilian PAs. In addition, our study illustrates the most common biodiversity knowledge shortfalls in the tropics.

## Introduction

As a megadiverse country, Brazil is a conservation priority. Large vegetation remnants still covering roughly 60% of the country^1^ house the largest share of Earth’s species^2^ in a variety of ecosystems ranging from tropical dense forests, dry forests, wetlands, savannas, to grasslands. To increase protection of these diverse ecosystems under a mounting threat of deforestation^3^, Brazil has invested heavily in expanding the network of protected areas (PAs), which includes three major categories of conservation units: strictly protected; sustainable use; and indigenous lands^4^. Despite the pivotal role of the country in global biodiversity conservation, the relevance and breadth of Brazil’s PAs for biodiversity conservation remain poorly known.

Brazil has been the target of several conservation assessments, including those reported in the 2005’s special edition of Conservation Biology that laid out the major challenges for future studies^5^. At that time, however, there were not much data available, which made spatially-explicit quantitative assessments difficult^5–9^. To make matters worse, taxonomic groups such as arthropods were poorly represented, which further hindered an in-depth analysis of their conservation status^9^. More than ten years later, this picture is rather different. A significant increase in records on species distribution due to online databases (e.g., GBIF, SpeciesLink, and Herpnet) are enabling pioneer countrywide assessments^10,11^. Although these studies need to cope with substantial knowledge gaps and sampling bias, methods have been developed to model the influence of these issues on biodiversity conservation analyses^11^ and hence point out regions with knowledge shortfall, which are essential to plan inventories^10,11^. Indeed, large gaps in biodiversity collection is common in Brazil, and more often than not the norm for tropical countries^12–17^. Yet most studies on conservation gaps in PAs disregard this shortfall.

Previous biodiversity assessments in Brazil, and in other countries, focused on specific taxonomic groups (*e.g*. birds, amphibians) as surrogates for the whole biota^18–21^. However, since sampling biases and biological knowledge shortfalls tend to affect equally all taxa, there is little empirical evidence supporting the representativeness of surrogates in large tropical countries^11^. In addition, some groups are systematically neglected in conservation analysis—*e.g*. arthropods^9,22,23^, despite their relevant ecosystem services, such as pollination^24,25^. Comprehensive biodiversity studies need therefore to include as many taxa as possible.

The majority of conservation studies have evaluated species distribution and threats to their maintenance only, and thus disregarded the evolutionary content of species assemblages^18,20,26^ in spite of the relevance of phylogenetic information for targeting areas for conservation^27^. This has been, in large part, due to the paucity of phylogenetic hypotheses for the Brazilian biota^27,28^. Nevertheless, this panorama is changing. Recent advances in molecular data acquisition led to a remarkable surge in publications on phylogenies of tropical species^28^, which are beginning to enable the use of evolutionary lineages in regional conservation studies^29^. Building upon this line of research, here we apply a wide compilation of phylogenetic trees together with a large database on species distribution encompassing vertebrate, arthropod, and angiosperm groups to assess biodiversity conservation and knowledge gaps in the Brazilian PAs.

## Results

**Figure 1:**
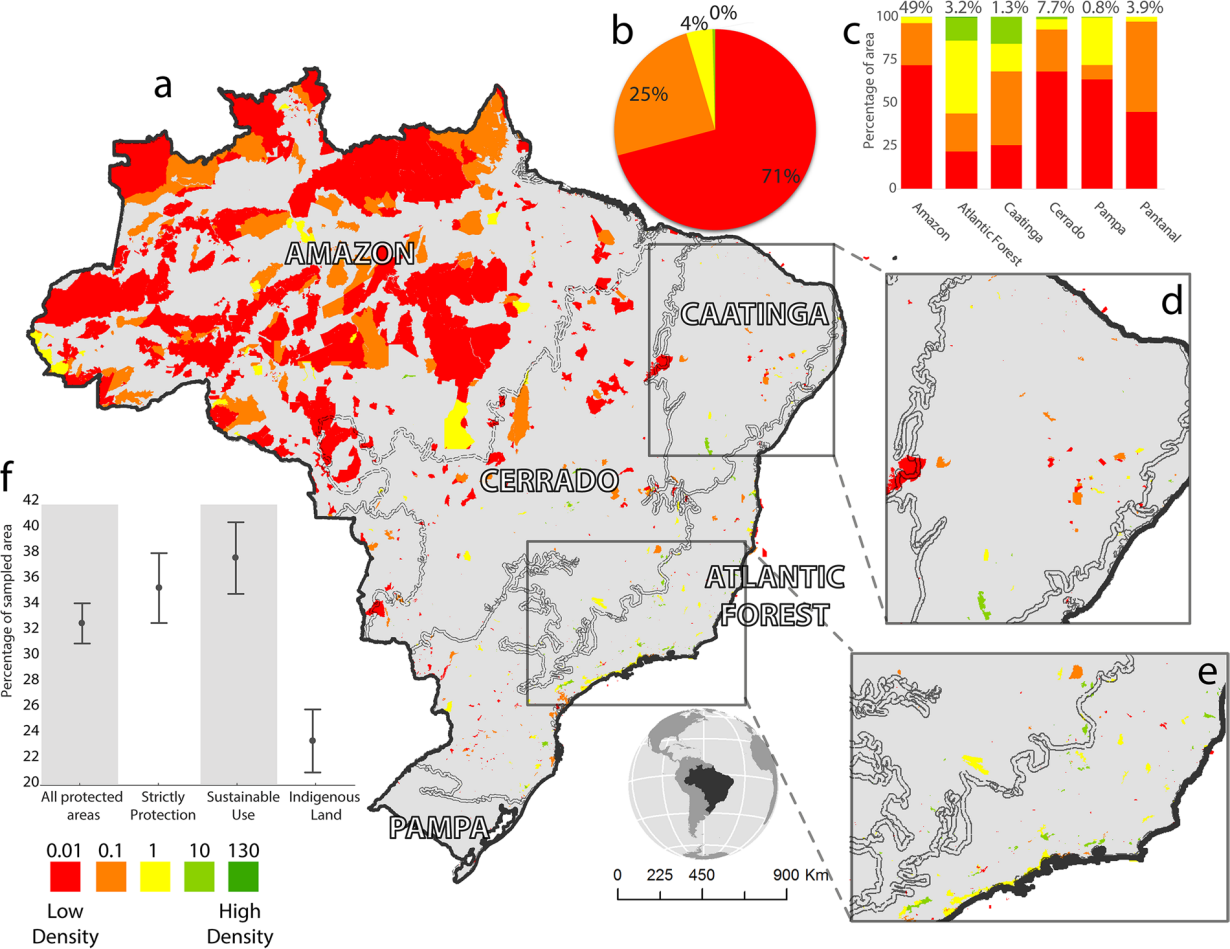
Density of species distribution records within PAs in Brazil (**a**). The pie chart (**b**) shows the percentage of the Brazilian territory within each record density category (number above legend represents the number of records per km^2^). The bar graph (**c**) shows the areal percentage per record density class within each Brazilian biome. Numbers above the bars represent the areal percentage of each biome covered by PAs. The insets show the Caatinga (**d**) and the southeastern Atlantic Forest (**e**), which are covered by particularly small conservation units. The lower graph (**f**) shows the average and standard deviation of the sampled area in each PA category. *Map created in ArcGIS 10.1* (http://www.esri.com).

The Brazilian PA network currently encompasses 1,743 units covering roughly 25% of Brazil’s territory and protecting 39% of the remaining area of native vegetation. The Amazon biome houses the largest extent of PAs (49%, Fig. 1a–c), Cerrado has 7.7%, whereas the others biomes (Atlantic Forest, Caatinga, Pampa and the Pantanal) contain less than 4% of Brazil’s PA geographic coverage (Fig. 1c).

Less than 1% of PAs are well sampled, with 10 to 130 records per km^2^ (Fig. 1a,b and Figure S1). More densely sampled PAs are found in the Atlantic Forest biome and in the Caatinga biome, respectively in the southeast and the northeast of the country (Fig. 1c,d,e). By contrast, 71% of PAs have low sampling density with 0 to 0.01 species record per km^2^ (Fig. 1a,b). PAs of the Atlantic Forest and the Caatinga are proportionally better sampled than those of the Amazon, Cerrado, Pampa and Pantanal (Fig. 1c). Atlantic Forest, Caatinga, Cerrado, Pampa, and Pantanal present no significant difference in record density inside and outside PAs (H = 0.2412 p = 0.8093 H = 1.6579 p = 0.0973, H = 1.0500 p = 0.2937, H = 0.2864 p = 0.7745, H = 0.6014 p = 0.5475, respectively), while in the Amazon there is a significantly higher record density within PAs (H = 6.3701 p = 0.0001).

**Figure 2:**
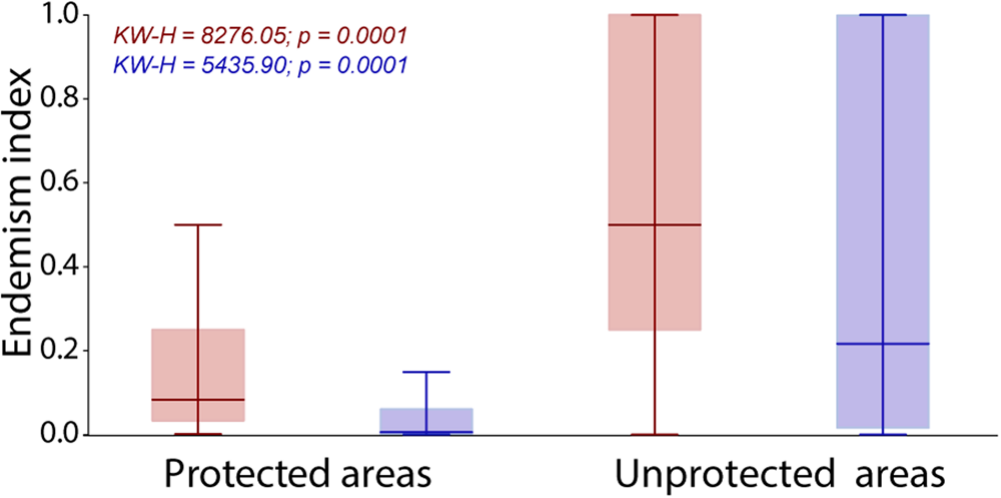
Median and percentiles (25–75%) of the index of endemism (WE) of the species within protected and unprotected areas. The blue bars show values for the sampling effort-corrected WE index.

Our results show that about 50% of total number of PAs is not even sampled with at least one species occurrence record. Sustainable Use protected areas hold the highest mean percentage of sampled area (37%), while strictly protected areas contain 35%, and indigenous lands only 23%, (Fig. 1f). Overall, PAs protect species with a median Weight Endemism index (WE) of less than 0.1, while the median of WE index for species outside PAs is above 0.4 (Figs 2 and S2). A similar pattern was observed for the sampling effort-corrected WE index, which includes the uncertainty of species distribution (with median below 0.005 in PAs and above 0.2 in unprotected areas).

**Figure 3:**
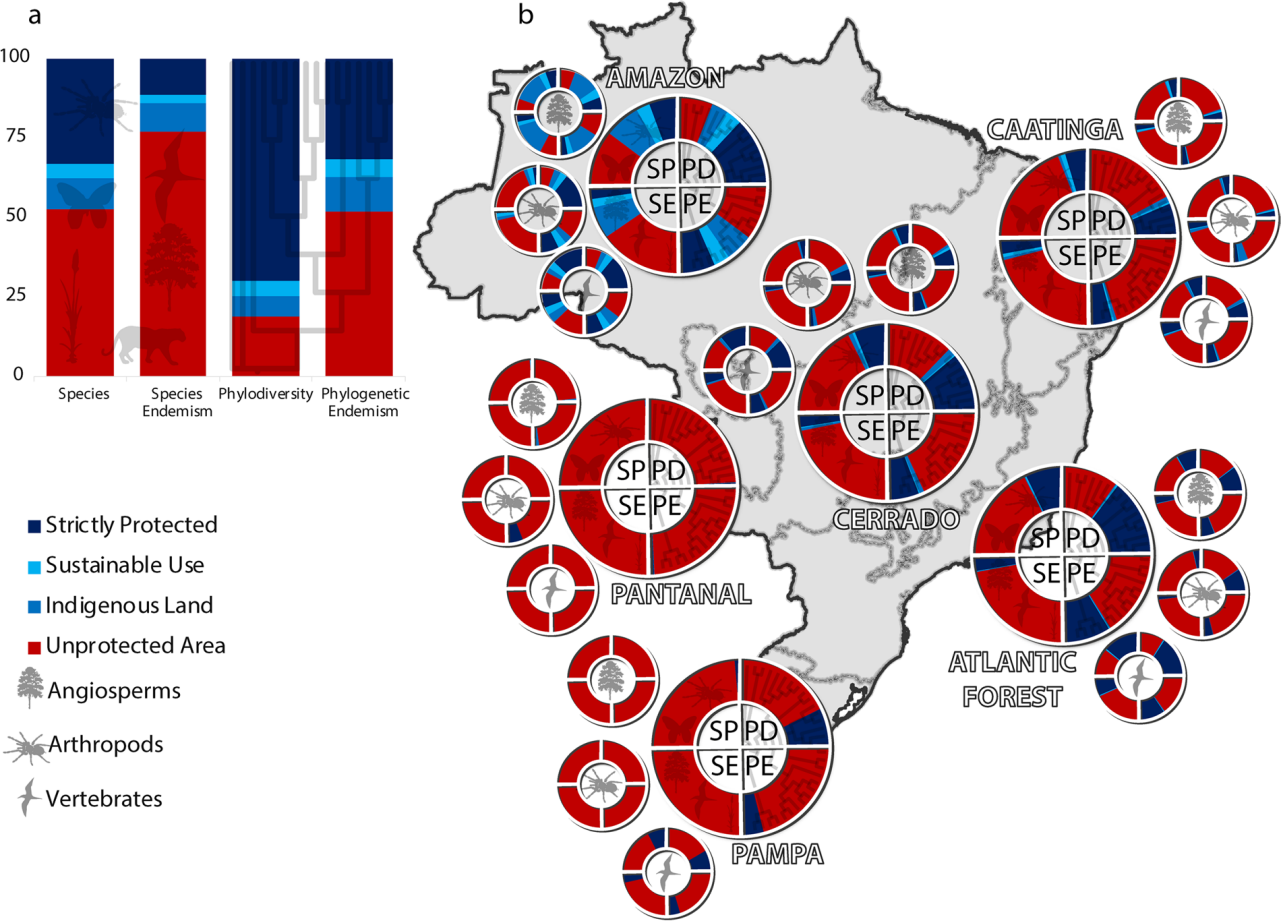
Proportion of protection for each dimension of biodiversity (species, endemism, phylodiversity and phylogenetic endemism) inside each PA category. The endemism is the proportion of the sum of the index of endemism of all Brazilian species and lineages analysed. Results are shown for the entire Brazilian territory (**a**) and for each Brazilian biome (**b**). Each circular chart indicates a biodiversity dimension inside each quadrant (SP = species, PD = phylogenetic diversity, SE = species endemism, PE = phylogenetic endemism. The smaller graphs show the results for each taxonomic group separately (angiosperms, arthropods and vertebrates). *Map created in ArcGIS 10.1* (http://www.esri.com).

Unprotected areas comprise 56% of the species and 38% of the lineages included in our database (Fig. 3). Roughly, 77% of the sum of WE values of all the Brazilian species and 56% of the sum of the phylogenetic endemism index occur outside PAs (Fig. 3). Most of the species and lineages found inside PAs are located in strictly protected conservation units, which also contain the highest percentage of the sum of the indices of endemism and phylogenetic endemism (Fig. 3). All biomes show a similar pattern, although the Pampa and the Pantanal contain most species, lineages and endemism (of species and lineages) outside PAs due to the low PA coverage (Fig. 3). Conversely, the Amazon contains most species, lineages and endemism (of species and lineages) inside PAs (Fig. 3). These results were generally congruent between taxonomic groups (Fig. 3), although vertebrates had the highest proportion of species, evolutionary lineages, endemism and phylogenetic endemism inside PAs (Fig. 3).

**Figure 4:**
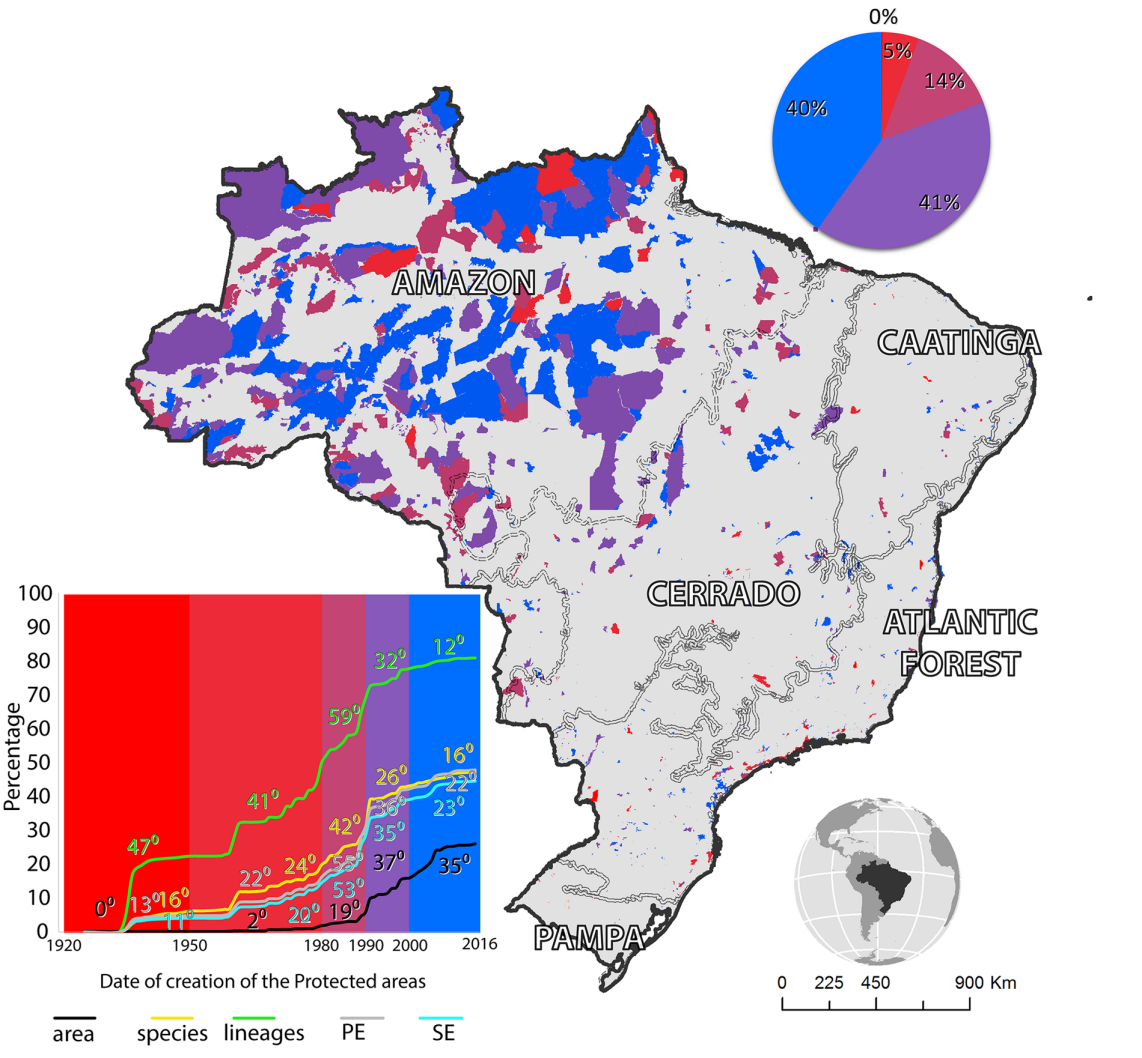
The increment in PA and biodiversity protection in Brazil over time. PAs are categorized according to their date of creation. The numbers on the curves indicate the slope of the time interval in degrees. Colors represent the same time intervals on the chart and on the map. PE: Phylogenetic endemism, SE: Species endemism. *Map created in ArcGIS 10.1* (http://www.esri.com).

About 80% of the current Brazilian PAs were designated after 2000 (Fig. 4). This expansion resulted in an increase in protected species, lineages, endemism and phylogenetic endemism (Fig. 4). However, the increment in biodiversity protection, measured by using all analysed biological dimensions, has slowed down since 2000, even though this was the period that PAs expanded most (Fig. 4).

**Figure 5:**
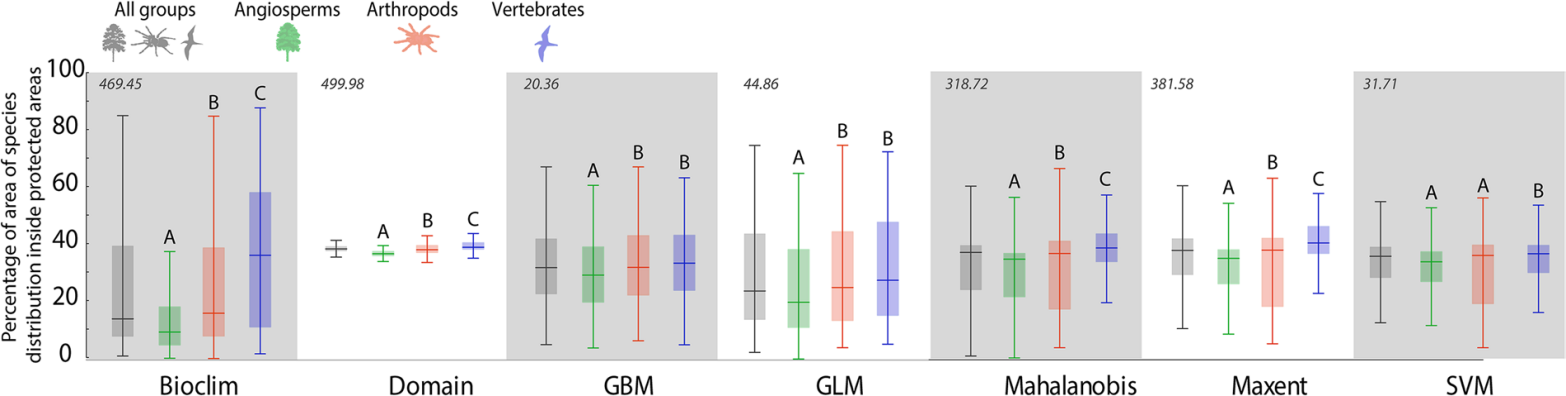
Median and percentiles (25–75%) of the percentage of species ranges inside PAs, as estimated by species distribution models. The numbers above indicate the value of Kruskall-Wallis’ H. The letters indicate the significant differences between the taxonomic groups in each algorithm.

Finally, all SDMs (Bioclim, Domain, GBM, GLM, Mahalanobis, Maxent and SVM) indicate that less than 40% of the (median) estimated species distribution area falls within PAs. However, most of estimated species distribution area are below 30% (median value). None of the species geographic distribution inside PAs has percentiles above 50%, except the ones of vertebrates in Bioclim model. Taxonomic groups (angiosperms, arthropods and vertebrates) show significant differences in the Kruskall-Wallis test regarding representativeness estimates in PAs (Fig. 5).

## Discussion

Our results show that biodiversity knowledge inside the Brazilian PAs remains scant. This shortfall is a hurdle to effectively managing PAs, especially given the scarcity of infrastructure and personnel^30^. While this knowledge shortage can be partially ascribed to the fact that many PAs were designated only recently, it also ensues from the lack of studies to support their creation and management. Rather than being a specificity for Brazil, these deficiencies are a common problem for most tropical PAs^12,14^.

There is a great need for further biodiversity inventories within the Brazilian PAs. Our results based on species occurrence points indicate that PAs protect a considerable part of the known Brazilian biodiversity, encompassing roughly half of the species and phylogenetic endemism, and much of the phylodiversity. This is remarkable, especially considering the low sampling in PAs and their low geographic coverage outside the Amazon. Indigenous lands in particular play an important role in biodiversity protection, as well as climate change mitigation^4^ given that these areas strongly deter deforestation^31^. In this regard, relaxation of Brazilian environmental laws to allow mining within PAs^32^ poses a threat to their rich biodiversity, which still remains only scarcely mapped.

Brazilian PAs, however, are apparently not protecting most endemic species and lineages. Species outside PAs were, on average, more restricted in distribution than those occurring within PAs (Fig. 2). This could be due to bias in our knowledge on species endemism^11^, especially considering that most PAs are poorly sampled. However, sampling effort does not differ between the majority of PAs and unprotected areas. In this case, results also indicate the same pattern, with endemic species distribution better represented outside PAs. This is particularly worrisome given that endemic species and lineages are more susceptible to extinction^33^ in the face of climate change^34,35^. In sum, species recorded in our database are poorly represented inside the Brazilian PAs, considering both the empirical knowledge on species distribution and the results of predictive models (SDMs). Yet we must account that both methods are affected by sampling bias and thus present an incomplete view of the biodiversity realm. This problem equally affects all taxonomic groups, including vertebrates and angiosperms, whose distribution is better known than that of arthropods^22^.

Although the arthropod protection gap is usually attributed to the lack of knowledge on this group^22^, evidences indicate that knowledge shortfalls are more similar between vertebrates, angiosperms and arthropods than anticipated^11^. Even though different groups have similar sampling problems, they do not present the same geographic distribution pattern^11^. These results speak against the use of taxonomic surrogates for planning conservation priorities based on the argument that a group (e.g. vertebrates) has a better-known geographic distribution than those of other groups. Because much more data on invertebrate diversity and distribution are now available than ten years ago^9^, we argue that there is no justification for the use of a few taxa as surrogates for the entire biodiversity spectrum. Conservation prioritization assessments should therefore rely on analyses that encompass most information on vertebrates, invertebrates and plants to effectively protect biodiversity.

Our results show that the expansion of PAs before the 2000s is closely related to the increase in the protection of biodiversity. On the other hand, the recent expansion in PAs in Brazil has not resulted in a comparable increase in biodiversity protection (Fig. 5). This may be related to low sampling effort in the recently designated PAs as well as in the remainder of the country^11^. Poorly sampled PAs should therefore be a priority for biodiversity inventories. This is particularly important given that these PAs may be protecting unknown biodiversity. Furthermore, special attention should be paid to evolutionary lineages as they may have a different distribution from that of species. In fact, our results show that the evolutionary lineages with more restricted distribution occur in a greater extent outside PAs. These lineages are fundamental to understanding the evolutionary processes underpinning biodiversity^27^, and thus should be considered in planning conservation priorities. Despite the increase in the availability of phylogenetic data over the last decade^28^, we still know little about the evolutionary history of the Brazilian species, since our phylogenetic compilation embraces only 14% of the species in our database. Thus, expanding the number of phylogenetic studies is fundamental for advancing the use of evolutionary lineages for planning conservation^27^.

Although PAs in most Brazilian biomes show the same deficit in biodiversity protection, we observed a few important differences. PAs in the Pampa and the Pantanal protect the lowest percentage of species and lineages. This is due to the small geographic coverage of PAs within these biomes^36^. This calls for urgently designating new PAs to counteract the potential impact of agricultural expansion on biodiversity of these regions^36–38^. The same is true for the Cerrado, the most coveted biome for agribusiness expansion^1,36,39,40^. Aside from the Amazon, all Brazilian biomes have a PA coverage well below the 17% recommended by the Biodiversity Convention^41^, thus reinforcing the need for new PAs. Moreover, most of the Amazonian PAs are poorly known and need proper implementation infrastructure^30^. Additional efforts like the ARPA program (programaarpa.gov.br) should be encouraged to ensure conservation of the large expanses of the Amazon PAs.

In conclusion, our knowledge on biodiversity both within and outside Brazilian PAs remains scant. In this regard, the countrywide panorama presented herein is a contribution to design further inventories in order to map and secure the biodiversity of Brazilian PAs. Rather than a specific case for Brazil, this is a common context for the tropics^42^. Opportunities other than biodiversity priorities often drive the creation of PAs worldwide^43^. Regardless of the policy mechanisms behind PA designation^44^, biodiversity inventories and analyses should be a priority in their implementation. These analyses must encompass most groups and lineages as well as sampling uncertainty to effectively map conservation priorities.

## Material and Methods

### Dataset

We built a large database on Brazil’s biodiversity comprising 882,468 distribution records of vertebrate (birds, mammals and anurans), arthropod (bees, spiders, millipedes, Orthoptera, dragonflies, moths and Diptera) and angiosperm (Asteraceae, Bromeliaceae, Fabaceae, Melastomataceae, Myrtaceae, Orchidaceae, Poaceae, and Rubiaceae) species. We compiled occurrence data from the following online databases: GBIF (http://www.gbif.org); SpeciesLink (http://www.splink.org.br); Birdlife International (http://www.birdlife.org), Herpnet (http://www.herpnet.org), Nature Serve (http://www.natureserve.org); and Orthoptera Species File (ortho-ptera.speciesfile.org). Access date is December 2014. The names of the taxonomic groups and the location filter “Brazil” were used in all the search queries. We also compiled data contained in the taxonomic literature and biodiversity inventories(Appendix S2).

### Phylogenetic tree reconstruction

In order to identify geographic patterns of evolutionary lineages, we compiled phylogenetic trees of taxa with geographic distribution limited to Brazil (Figure S3). The trees were obtained from peer-reviewed articles that address phylogenies of Brazilian species and from a supertree built upon a review of the Tree of Life^28^ (Appendix S1 and Figure S3). This supertree was pruned to cover only Brazilian species. All trees were merged into a supertree by using a matrix representation with parsimony implemented in package phytools in R^45^. We estimated the supertree by using the method proposed by Schliep^46^ that employs the parsimony ratchet method^47^. The phylogenetic trees obtained from peer-reviewed articles were converted to the newick format using TreeSnatcherPlus software^48^. Since branch lengths are not comparable between different trees and occasionally are not even available, we assumed all branches lengths equal to one.

### Conservation and sampling effort gaps

To identify the collection effort within PAs, we quantified the number and density of species distribution records per PA, based on a PA map (http://maps.csr.ufmg.br/). We categorized the Brazilian territory into unprotected and the three classes of PAs: Strictly protected conservation units whose primarily goal is the preservation of biological diversity; sustainable use reserves, which seek to balance conservation with sustainable use of the natural resources; and indigenous lands that provide environmental, social and cultural sanctuaries to indigenous groups^4^. We also compared the extent of PAs in each Brazilian biomes (http://mapas.mma.gov.br/). To test whether the knowledge on species distribution within PAs is different from that observed outside them, we compared the record density distribution within and outside PAs using 0.5° hexagonal grid cells as sampling units for the Kruskal-Wallis test in R software 3.3 (http://www.r-project.org). To identify the proportion of sampled area within PAs, we estimated the sampled area within each PA using a 1-km buffer around each species occurrence record. We used a 1-km radius because this value corresponds to the mode of maximum collection distances observed in Brazil^11^.

### Biodiversity gaps in conservation

To identify the degree of biodiversity protection throughout the country, we estimated the proportion of species, evolutionary lineages, endemism and phylogenetic endemism inside and outside of PAs. We also estimated the species and phylogenetic endemism inside each PA as follows: (1) for protection of evolutionary lineages, we summed the lengths of tree branches connected to species present within each PA; and (2) for protection of endemic species, we chose the weighted endemism index (WE)^49^, which expresses endemism as an inverse function of the species range. Thus, species with restricted distribution have values near one. Since we do not have data on branch lengths and as a result had to assume the same length for all of them, we cannot infer evolution along the branches. This index was derived from a grid of 0.5° hexagonal cells. Species that occur in a single hexagon have the maximum value of the index. We chose this unit size to avoid assuming that species occur in very large areas (in the case of the choice of larger grid cells) or that they occur only in very small areas (in the case of smaller grid cells). Since the estimated distribution of any species is inherently uncertain, we controlled for this uncertainty by using an approach that obtains a sampling effort-corrected WE index, below (Eq. 1):

1 $$\frac{A\ast B}{((A\ast B)+((1-A)\ast (1-C)))}\,$$

A = WE based on distribution records, as aforementioned. B was calculated using the product of the WE and the sampling effort, which was expressed as the record density. The density of records is the average kernel index estimated from a buffer of 50 km around each occurrence point of each species analysed. C is obtained using the number of species sampled, so that more than 150 species records is tantamount to 0.999. The maximum value was set at 150 because the distribution frequency of records per species reaches an asymptote at this value. This asymptote represents the rare cases of species with relatively more records (Figure S1). Species with fewer records were assigned in a linear manner lower values up to 0.00001 for species with only one record. A, B and C values were between but not equal to 1 or 0.

To quantify the protection of endemism, we summed the indices of each hexagon within PAs and unprotected areas to compare the protection percentage. In order to verify if the presence of endemism is different inside and outside PAs, we compared the median values using a Mann-Whitney test because the number of samples (species) varies across the units of analysis (classes of PAs). We quantified phylogenetic endemism using the Phylogenetic Weighted Endemism Index (PWE)^50^, based on 0.5° hexagons. This index is similar to WE, but in this case the lineage endemism is equivalent to the sum of branch lengths connected to each species.

The geographic coverage of PAs in Brazil expanded significantly over the last 15 years. To test the effectiveness of this expansion on biodiversity protection we generated species, lineage, endemism and phylogenetic endemism accumulation curves through time. The slope of each curve was analysed using a linear model over time-intervals defined by breakpoints of PA designation phases: 1903 to 1950; 1951 to 1980; 1981 to 1990; 1991 to 2000; and 2001 to 2016.

### Representativeness of PAs

To estimate the efficacy of PAs to protect species, we calculated the areal percentage for each species distribution within PAs. Since a considerable portion of the Brazilian native vegetation has been removed, we estimated this percentage considering only remnants of native vegetation (http://maps.csr.ufmg.br/). We estimated species distribution through seven species distribution models (SDM) algorithms: Bioclim; Domain; Mahalanobis distance; Maxent, Generalized Linear Model (GLM); Generalized Boosting Model (GBM or Boosted Regression Trees); and Support Vector Machine (SVM)^51^. Since the spatial prediction of SDMs may be influenced by the number and geographic accuracy of records^52^, we selected the 4,344 species with more than 15 accurately geo-referenced occurrence points (for more details see appendix 3 in Oliveira *et al*.^11^). SDMs were based on the first four axes of a PCA correlation matrix performed using 19 bioclimatic (http://www.worldclim.org/) and two topographic variables (elevation from Worldclim and slope derived from elevation data in ArcGIS 10.1). We used the lowest suitability value in training points as a threshold because it is based exclusively on empirical evidence, *i.e*. our samples. Thus, the adoption of this threshold is a conservative approach because it minimizes the limits considered as suitable for the species to the range of the empirical data. The PCA was implemented in ArcGIS using 5-km cell resolution. To exclude models that presented random predictions, we used the area under the curve (AUC) through pseudo absences. We used only SDMs that presented an AUC greater than 0.7. The species distribution estimated from SDMs and the one from interpolation of records were overlaid on PA maps to estimate the representativeness of species distribution, *i.e*. percentage of the estimated area of distribution of species contained in PAs. Spatial analyses were performed using ArcGIS and Dinamica EGO^53^. To test differences between taxonomic groups (angiosperms, arthropods and vertebrates) in each model, we used the Kruskal-Wallis test and multiple comparisons of ranks.

## Electronic supplementary material

Appendix S1

Appendix S2
