# Publisher Correction: Biodiversity conservation gaps in the Brazilian protected areas

**DOI:** 10.1038/s41598-018-22953-y

**Published:** 2018-03-19

**Authors:** Ubirajara Oliveira, Britaldo Silveira Soares-Filho, Adriano Pereira Paglia, Antonio D. Brescovit, Claudio J. B. de Carvalho, Daniel Paiva Silva, Daniella T. Rezende, Felipe Sá Fortes Leite, João Aguiar Nogueira Batista, João Paulo Peixoto Pena Barbosa, João Renato Stehmann, John S. Ascher, Marcelo Ferreira de Vasconcelos, Paulo De Marco, Peter Löwenberg-Neto, Viviane Gianluppi Ferro, Adalberto J. Santos

**Affiliations:** 1 Centro de Sensoriamento Remoto, Instituto de Geociências, Universidade Federal de Minas Gerais – UFMG, Av. Antonio Carlos 6627, CEP 31270-901 Belo Horizonte, MG Brazil; 2 Departamento de Zoologia, Instituto de Ciências Biológicas, Universidade Federal de Minas Gerais – UFMG, Av. Antonio Carlos 6627, CEP 31270-901 Belo Horizonte, MG Brazil; 3 Departamento de Biologia Geral, Instituto de Ciências Biológicas, Universidade Federal de Minas Gerais – UFMG, Belo Horizonte, MG Brazil; 4 Laboratório Especial de Coleções Zoológicas, Instituto Butantan, São Paulo, SP Brazil; 5 Departamento de Zoologia, Universidade Federal do Paraná, Curitiba, Paraná Brazil; 6 Instituto Federal Goiano – IFGoiano, Departamento de Biologia, Urutaí, Goiás Brazil; 7 Sección Palentología de Vertebrados Museo Argentino de Ciencias Naturales “Bernardino Rivadavia” Avenida Angel Gallardo 470, C1405DJR Buenos, Aires, Argentina; 8 Laboratório Sagarana, Instituto de Ciências Biológicas e da Saúde, Universidade Federal de Viçosa – UFV, Campus Florestal, Florestal, MG Brazil; 9 Departamento de Botânica, Instituto de Ciências Biológicas, Universidade Federal de Minas Gerais – UFMG, Belo Horizonte, MG Brazil; 10 Department of Biological Sciences, National University of Singapore, Singapore, Singapore; 11 Instituto Prístino, Rua Santa Maria Goretti, 86, Barreiro, CEP 30642-020 Belo Horizonte, MG Brazil; 12 Departamento de Ecologia, Instituto de Ciências Biológicas, Universidade Federal de Goiás, Goiânia, Goiás Brazil; 13 Universidade Federal da Integração Latino-Americana, Foz do Iguaçu, PR Brazil

Correction to: *Scientific Reports* 10.1038/s41598-017-08707-2, published online 22 August 2017

The original version of this Article contained a typographical error in the spelling of the author Daniel Paiva Silva, which was incorrectly given as Dasniel Paiva Silva. This has now been corrected in the PDF and HTML versions of the Article.

In addition, the Acknowledgements section in this Article was incomplete.

“This work was supported by the Climate and Land Use Alliance, Conselho Nacional de Desenvolvimento Científico e Tecnológico, Fundação de Amparo à Pesquisa do Estado de Minas Gerais, and the Humboldt Foundation. U. Oliveira was financially supported by a CNPQ (437209/2016–4), A.J. Santos was sponsored by research grants from CNPq (475179/2012-9, 407288/2013-9, 306222/2015-9), FAPEMIG (PPM-00335-13, PPM-00651-15) and Instituto Nacional de Ciência e Tecnologia dos Hymenoptera Parasitóides da Região Sudeste Brasileira (http://www.hympar.ufscar.br/). CJB de Carvalho (CNPq 304713/2011-2), AD Brescovit (CNPq 303028/2014-9; FAPESP 2011/50689-0), Data acquisition on stingless bee specimens at the American Museum of Natural History by JSA occurred with help the from HH Go, A Pfister, M Tuell and ES Wyman. It was supported by RG Goelet and by a NSF-DBI grant (#0956388 with JS Ascher as the P.I., and JG Rozen Jr. and D Yanega as co-P.I.s).”

now reads:

“This work was supported by the Climate and Land Use Alliance, Conselho Nacional de Desenvolvimento Científico e Tecnológico, Fundação de Amparo à Pesquisa do Estado de Minas Gerais, and the Humboldt Foundation. U. Oliveira was financially supported by a CNPQ (437209/2016–4), A.J. Santos was sponsored by research grants from CNPq (475179/2012-9, 407288/2013-9, 306222/2015-9), FAPEMIG (PPM-00335-13, PPM-00651-15) and Instituto Nacional de Ciência e Tecnologia dos Hymenoptera Parasitóides da Região Sudeste Brasileira (http://www.hympar.ufscar.br/). CJB de Carvalho (CNPq 304713/2011-2), AD Brescovit (CNPq 303028/2014-9; FAPESP 2011/50689-0), Data acquisition on stingless bee specimens at the American Museum of Natural History by JSA occurred with help the from HH Go, A Pfister, M Tuell and ES Wyman. It was supported by RG Goelet and by a NSF-DBI grant (#0956388 with JS Ascher as the P.I., and JG Rozen Jr. and D Yanega as co-P.I.s). DPS received a research productivity grant from Federal Goiano Institute (Bolsa PAPPE process number 23216.000573/2017-61).”

