# Supplementary material for: Biodiversity conservation gaps in the Brazilian protected areas
Source: Sci Rep. 2017 Aug 22;7:9141. doi: 10.1038/s41598-017-08707-2 (PMC5567310; doi:10.1038/s41598-017-08707-2)
Supplement: Supplementary file 1 — Appendix S1 [file 41598_2017_8707_MOESM1_ESM.pdf]

Appendix S1

Biodiversity conservation gaps in the Brazilian protected areas

Ubirajara Oliveira, Britaldo Silveira Soares-Filho, Adriano Pereira Paglia, Antonio D. Brescovit, Claudio J. B. de Carvalho, Daniel Paiva Silva, Daniella T. Rezende, Felipe Sá Fortes Leite, João Aguiar Nogueira Batista, João Paulo Peixoto Pena Barbosa, João Renato Stehmann, John S. Ascher, Marcelo Ferreira de Vasconcelos, Paulo De Marco, Peter Löwenberg-Neto, Viviane Gianluppi Ferro, Adalberto J. Santos

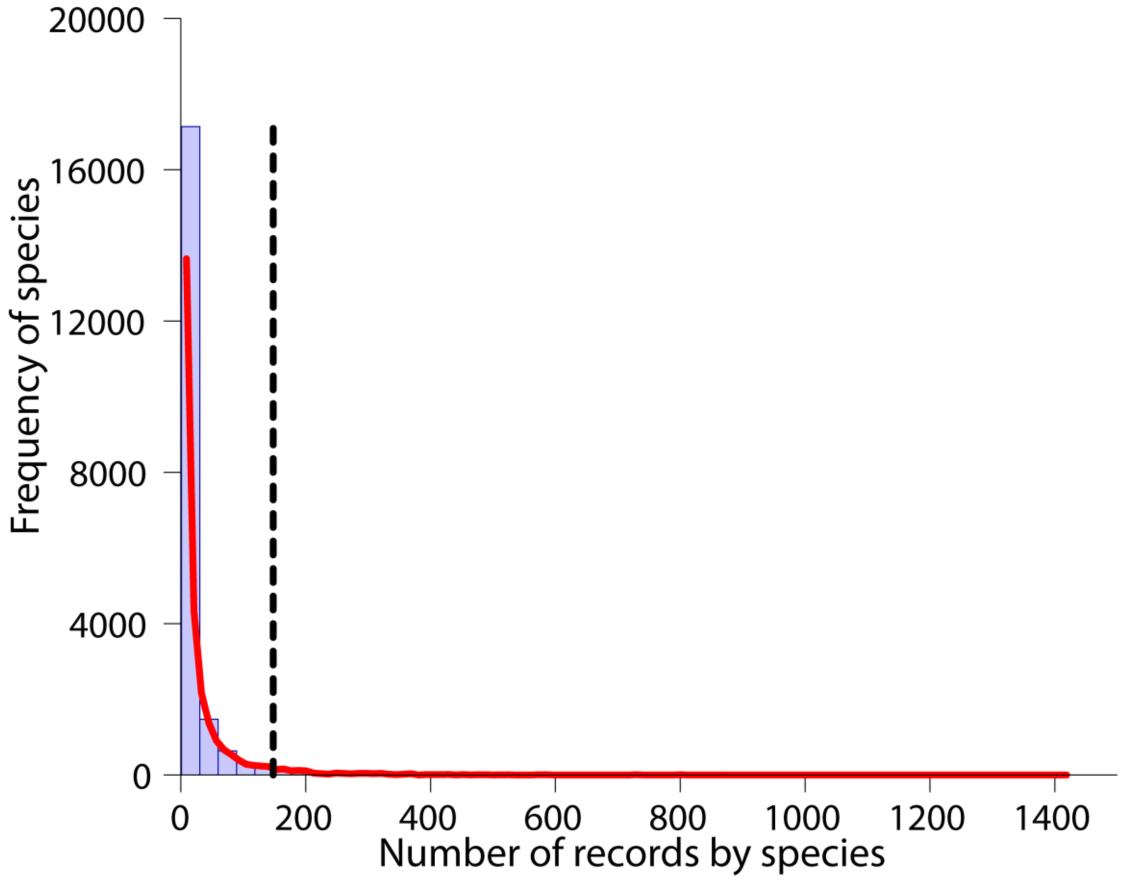

Figure S1: Frequency of distribution records of Brazilian species. The dashed line indicates the point of curve stabilization.

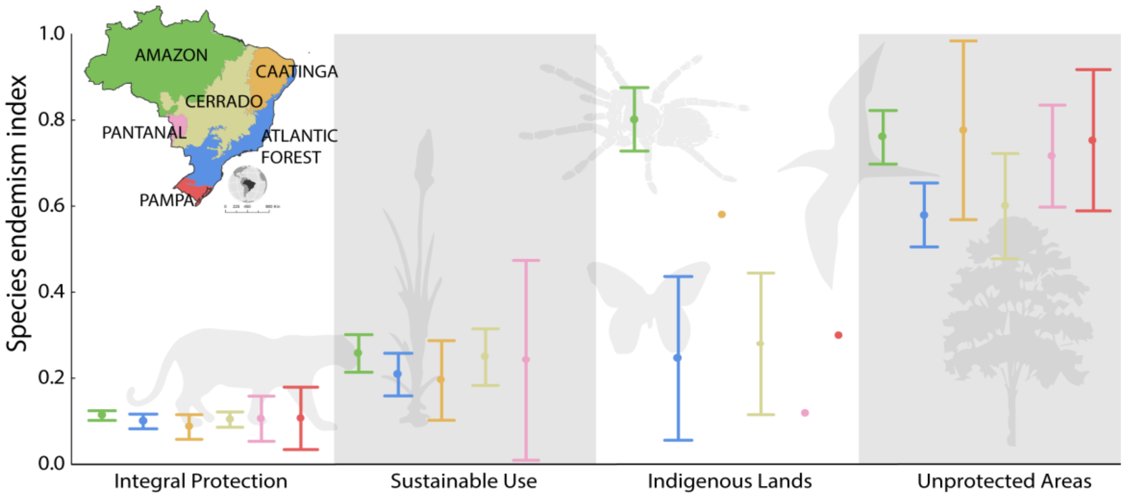

Figure S2: Mean and standard deviation of the index of endemism of the species within each classes of Brazilian PAs and in unprotected areas. Colours show the Brazilian biomes. *Map created in ArcGIS 10.1 (http://www.esri.com).*

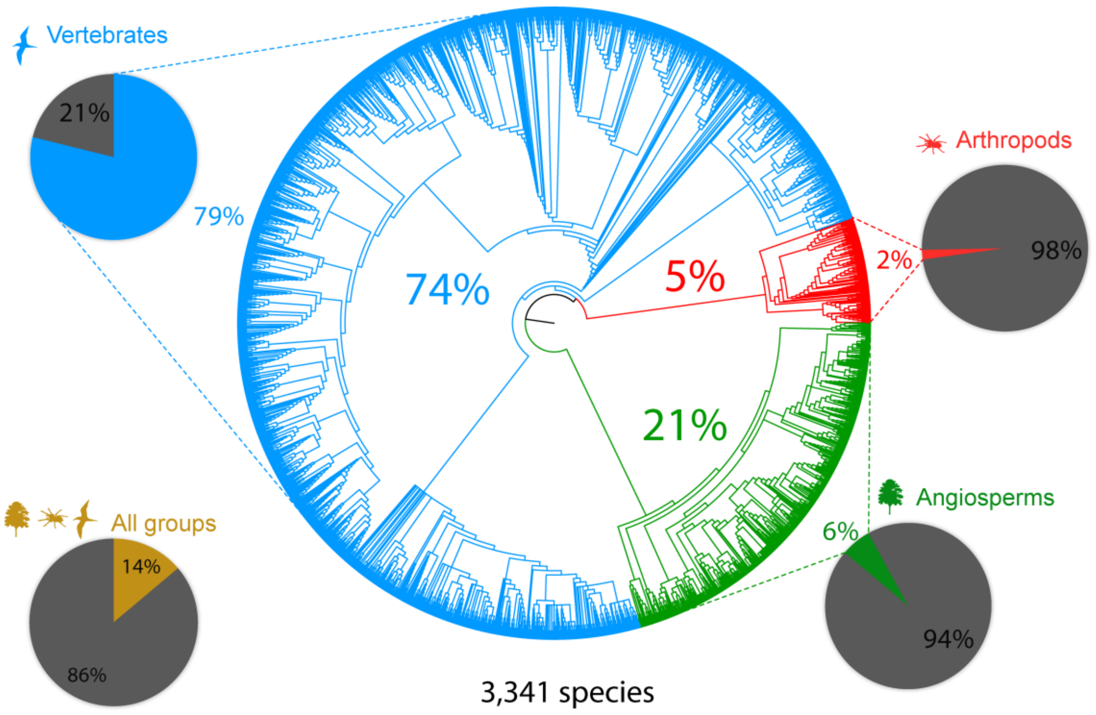

Figure S3: Phylogenetic supertree with 3,355 Brazilian species (Appendix S4). The pie charts show the proportion of species from the species distribution database represented on the supertree. The numbers in the tree represent the percentage of each group in the supertree.
